# Challenges in tuberculosis screening before TNF-α inhibitors: A case report highlighting gaps in current practice

**DOI:** 10.1016/j.jctube.2026.100629

**Published:** 2026-07-01

**Authors:** Aurelie J.F.Schroë, Marijke Rutten, Sami O.Simons

**Affiliations:** a Department of Respiratory Medicine, Imeldaziekenhuis, Bonheiden, Belgium; b Department of Respiratory Medicine, Maastricht University Medical Center+, Maastricht, the Netherlands

**Keywords:** Tuberculosis, Immunosuppression, screening, IGRA, TST, case report

## Abstract

We present the case of a 34-year-old Polish man with a history of axial spondyloarthritis and Crohn's disease, who presented with respiratory and neurological symptoms whilst on TNF-α inhibitors treatment. He subsequently developed a fulminant pulmonary tuberculosis (TB) with cerebral involvement. Treatment was complicated by a paradoxical reaction and respiratory failure requiring intensive care admission. This case highlights the diagnostic challenges associated with TB screening prior to immunosuppressive therapy and underscores the limitations of single-test screening protocols. It further emphasizes that, in immunocompromised patients, both IGRA and TST have reduced sensitivity, and a dual testing strategy with repeated, risk-based screening may improve detection in selected high-risk populations.

This case also demonstrates that tuberculosis in immunosuppressed patients can follow a fulminant course, and that early detection and timely initiation of appropriate therapy are essential determinants of clinical outcome.

## Highlights

• TNF-α inhibitor therapy carries a high risk of TB reactivation.
• Combining IGRA and TST may improve sensitivity in selected high-risk populations.
• Indeterminate IGRA results are common in immunosuppressed individuals and still indicate elevated TB risk.
• TB risk is dynamic; repeated, risk-based screening is essential during long-term immunosuppression.

## 1 Introduction

Tuberculosis (TB) remains a major global health concern, particularly among immunocompromised individuals. The use of tumor necrosis factor-alpha (TNF-α) inhibitors, such as infliximab, markedly increases the risk of reactivation of *Mycobacterium tuberculosis* infection, with a relative risk of 16.2 (95% CI, 14.6–18.0) for developing active TB compared with the general population [1]. Pre-treatment screening for TB infection (TBI) is recommended prior to the initiation of TNF-α blocking agents, and treatment of TBI prior to progression to active disease can reduce the risk of developing TB disease by up to 90% [2]. In spite of these recommendations, consensus on the optimal diagnostic approach in clinical practice is lacking, and protocols vary considerably between institutions and national guidelines.

We report a case of severe active disseminated TB in a patient with Crohn's disease and axial spondyloarthritis, highlighting diagnostic challenges and underscoring the critical importance of sensitive screening strategies and timely intervention.

## 2 Case report

### 2.1 Medical history

A 34-year-old Polish man presented to the emergency department with complaints of shortness of breath, hemoptysis and a swollen painful right lower leg.

His medical history included HLA-B27 positive axial spondyloarthritis and Crohn's disease, previously managed with adalimumab, mercaptopurine and later infliximab due to therapy failure. The last administered dose of infliximab was four months prior to presentation.

Prior to starting immunosuppressive therapy, an interferon-gamma release assay (IGRA) had been performed, which rendered a negative result. The patient had not received prior BCG vaccination. The decision not to perform a TST was based on the local screening protocol rather than concerns regarding reduced TST specificity due to Bacillus Calmette-Guerin (BCG) vaccination.

The patient reported he had been experiencing persistent headaches for a year. An outpatient neurological evaluation, including brain MRI, revealed multiple bilateral spherical T2-hyperintense lesions, raising suspicion of an opportunistic infection (Fig. 1). Diagnostic work-up was limited due to the patient's refusal of lumbar puncture.

**Fig. 1:**
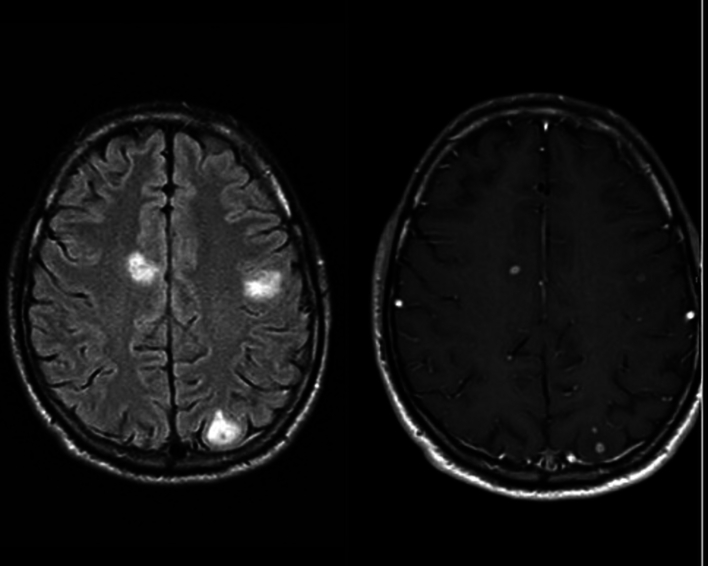
MRI cerebrum FLAIR (left) and with Gadolinium contrast (right) 2 months before admission to the emergency department showing bilateral diffuse T2 hyperintens round laesions surrounded by edema.

### 2.2 Investigations

On examination, the patient appeared cachectic. Vital signs showed a blood pressure of 90/50 mmHg, a heart rate of 100 bpm, respiratory rate of 20/min, oxygen saturation of 96% on room air, and a temperature of 37.8 °C. Cardiopulmonary examination was unremarkable. His right leg was tense, warm, and edematous.

Laboratory investigations revealed elevated inflammatory markers and markedly increased D-dimer levels (Table 1). A computed tomography (CT) of the chest was performed that demonstrated bilateral (sub)segmental pulmonary emboli, extensive centrilobular and perilymphatic nodules, consolidations, and bronchiectasis, predominantly in the upper lobes (Fig. 2). Doppler ultrasound confirmed a deep vein thrombosis in the right leg. Further evaluation revealed a positive sputum culture for *Mycobacterium tuberculosis*.

**Table 1:** Laboratory results at presentation.

	Patients results	Reference range
Hemoglobin	6,4 mmol/L	8,2–11,0 mmol/L
MCV	80 fL	80–100 fL
Thrombocytes	207 10E9/L	130–350 10E9/L
Leukocytes	14,0 10E9/L	3,5–11,0 10E9/L
D-Dimer	>10.000 μg/L	≤500 μg/L
Glucose	5,8 mmol/L	3,1–7,8 mmol/L
Sodium	128 mmol/L	135–145 mmol/L
Potassium	3,8 mmol/L	3,6–5,0 mmol/L
Calcium	1,93 mmol/L	2,10–2,55 mmol/L
Urea	4,6 mmol/L	3,0–8,0 mmol/L
Creatinine	62 μmol/L	60–115 μmol/L
eGFR CKD-EPI	>90 mL/min/1,73 m2	≥90,0 mL/min/1,73 m2
Alkaline phosphatase	499 U/L	≤115 U/L
γ-GT	111 U/L	≤55 U/L
AST	90 U/L	<35 U/L
ALT	55 U/L	<45 U/L
LD	497 U/L	<248 U/L
hsTnT	10 ng/L	≤14 ng/L
NT-proBNP	30,0 pmol/L	≤15,0 pmol/L
Ferritin	1.885 μg/L	30–400 μg/L
CRP	149 mg/L	≤10 mg/L
Albumin	16,6 g/L	32,0–47,0 g/L

(MCV: Mean Corpuscular Volume, eGFR CKD-EPI: estimated Glomerular Filtration Rate Chronic Kidney Disease Epidemiology Collaboration, γ-GT: gamma-Glutamyl Transferase, AST: Aspartate aminotransferase, ALT: Alanine aminotransferase, LD: Lactate dehydrogenase, hsTnT: high-sensitivity cardiac troponin T, NT-proBNP: N-terminal pro–B-type natriuretic peptide, CRP: C-reactive protein)

**Fig. 2:**
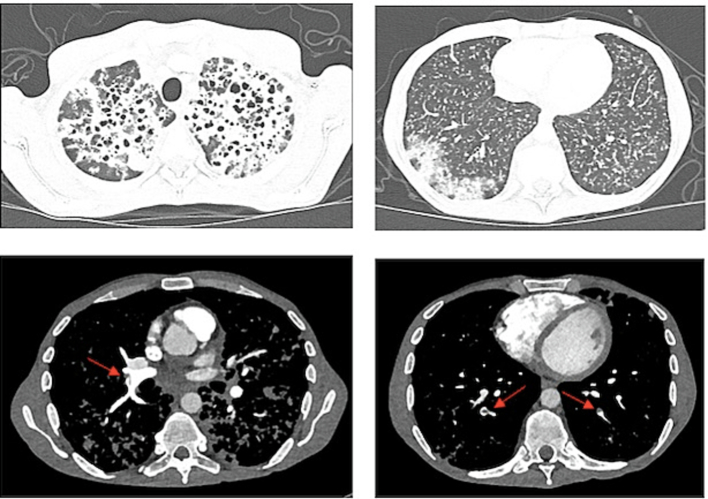
CT thorax at emergency department showing intrapulmonal centrilobular and perilymphatic nodules, pulmonary consolidations, bronchiectasis in the upper lobes and bilateral lung embolism (red arrows). (For interpretation of the references to colour in this figure legend, the reader is referred to the web version of this article.)

A repeated MRI showed partial regression of the cerebral lesions. A lumbar puncture was attempted but resulted in a dry tap, precluding cerebrospinal fluid analysis. The cerebral abnormalities were therefore considered possibly related either to infliximab-associated adverse effects with a differential diagnosis of tuberculomas in the context of active tuberculosis.

### 2.3 Treatment

A diagnosis of fulminant active pulmonary TB with a high suspicion of cerebral involvement was made and the patient was transferred to a specialized TB treatment center. Quadruple anti-tuberculosis therapy with rifampicin, isoniazid, pyrazinamide and ethambutol was initiated. Ethambutol was discontinued once drug susceptibility testing confirmed full sensitivity of the isolate, and pyrazinamide was stopped after completing two months of therapy. Anticoagulation with low-molecular-weight heparin (LMWH) was started for treatment of pulmonary embolism and deep vein thrombosis and continued for three months.

### 2.4 Follow up and outcomes

During treatment, the patient developed a respiratory insufficiency, with impending exhaustion despite high-flow oxygen supplementation and intensive care support. He had fever and high biochemical inflammation parameters consistent with a pulmonal paradoxical reaction with hyperinflammation. No other organs were involved. Adjunctive therapy with prednisolone in combination with anakinra was started. Because of no improvement under this therapy we eventually switched anakinra to infliximab. Under this therapy, his condition improved and he was discharged in stable condition.  He successfully completed nine months of anti-tuberculosis therapy at home and recovered well. Prednisolone was tapered off and Infliximab was subsequently continued for axial spondyloarthritis and Crohn's disease, with good therapeutic response.

A follow-up brain MRI obtained after completion of tuberculostatic therapy showed a significant decrease in the size of all intracranial tuberculomas, with only minimal residual lesions remaining, supporting an adequate response to anti-tuberculous therapy.

## 3 Discussion

### 3.1 TNF-α inhibitors and TB reactivation

This case illustrates the potentially severe consequences of tuberculosis reactivation in patients receiving immunosuppressive therapies. In patients receiving immunosuppressants, the incidence of TB disease is reported as 5.4 and 4.8 per 1000 person-years among those with positive TST and IGRA results, respectively [3], this is a relative risk of 16.2 (95% CI, 14.6–18.0) compared to the general population [1]. However, the risk of tuberculosis is not uniform across all immunosuppressive agents. TNF-α inhibitors, together with JAK inhibitors give the highest risk of TB reactivation [4]. TNF-α inhibitors increase susceptibility to both reactivation of latent infection and primary progressive TB due to the fact that these drugs impair the formation and maintenance of granulomas, essential for containing *Mycobacterium tuberculosis* [5]. Among TNF-α inhibitors, infliximab has consistently been associated with the highest risk of tuberculosis, whereas etanercept and certolizumab pegol appear to confer a lower risk [6]. Other immunosuppressants like IL12 and IL23 inhibitors, for example Ustekinumab, give a low risk of TB reactivation, nevertheless they might play a role in the formation of granulomas. The mechanism is not known [4]. Anti-CD 20 antibody, Rituximab, also has a low risk of TB reactivation which might be explained by its mechanism which does not affect the function of the T cells, the primary cells involved in protecting against active TB [4].

### 3.2 TB endemicity

In addition to the type of immunosuppressive therapy and the underlying disease, the local TB endemicity is a major determinant of the risk of developing TB, with significantly higher rates observed in TB-endemic regions [4].

Whether the present case resulted from reactivation of previously undetected latent tuberculosis infection or acquisition of a new infection remains uncertain. No documented tuberculosis exposure was identified after initiation of TNF-α inhibitor therapy. However, the patient frequently traveled between the Netherlands and Poland, his country of origin, where tuberculosis incidence remains higher than in the Netherlands, with an incidence of approximately 10 per 100,000 population [7]. This highlights the importance of careful assessment of travel history and other epidemiological risk factors, both prior to initiation of immunosuppressive therapy and during long-term follow-up.

### 3.3 Screening: single or dual testing strategy

Prior to initiation of immunosuppressive therapy, our patient underwent screening with an IGRA, which returned negative. A TST was not performed, in accordance with local and global guidelines. The World Health Organization (WHO) and the US Preventive Services Task Force, recommend screening for latent TBI with a single test - either IGRA or TST [8], [9]. The American College of Gastro-enterology advises that IGRA are likely to complement to TST and are preferred in patients with BCG vaccination in the past [10]. They suggest considering repeated TST testing in selected immunocompromised patients following an initial negative result, as part of a two-step testing strategy aimed at improving detection of latent tuberculosis infection [10]. A recent study stated that the developing TB under treatment with biological therapies is not completely preventable and neither dual testing nor periodic systematic re-screening for TB infection is warranted after a negative IGRA or TST [11].

However, other recent studies increasingly support a dual testing strategy (IGRA plus TST) over a single-test approach [4]. A large meta-analysis demonstrated that HIV positive individuals with either IGRA or TST positivity had significantly increased rates of progression to active TB compared to those with concordant negative results [3]. Similarly, data from a large Spanish registry showed a decrease of detected cases by respectively 40% and 24% when using IGRA or TST alone versus dual testing [12]. Together, these findings suggest that in high-risk populations a single negative result may be insufficient, and adding a second test could improve diagnostic sensitivity in selected high-risk populations.

### 3.4 Screening in immunocompromised population

In immunocompromised individuals, the sensitivity of both IGRA and TST is variable and generally lower than in immunocompetent populations [4], [13], [14]. The extent of this reduction depends on the cause and severity of the underlying immunosuppression [14].

Several alternative screening strategies have been proposed for patients scheduled to receive TNF-α inhibitors. Traditionally a TST is performed for screening before start of immunosuppressants. In populations originating from regions with a high tuberculosis burden, a positive TST is generally considered indicative of TBI regardless of previous BCG vaccination. In contrast, interpretation of TST results may be more challenging in low-incidence settings with high rates of BCG vaccination. Interferon-gamma release assays (IGRAs) have therefore emerged as an attractive alternative, particularly in BCG-vaccinated individuals, owing to their higher specificity. Nevertheless, neither TST nor IGRA can reliably exclude TBI [5].

IGRA results may occasionally be indeterminate, a phenomenon observed more frequently in immunocompromised patients. In the majority of such cases, this is attributable to failure of the positive control, reflecting insufficient IFN-γ production after mitogen stimulation. This occurs particularly in patients with severe immune dysfunction, such as those receiving TNF-α blockers, high-dose corticosteroids, or individuals with advanced HIV infection and low CD4 counts [13]. Importantly, patients with indeterminate IGRA results still carry a considerable risk of progression to active TB: approximately half the risk observed in IGRA-positive individuals and about three times higher than in those with negative IGRA results [13].

A combined TST and IGRA approach may increase sensitivity for detecting latent tuberculosis infection, as the two tests identify only partially overlapping populations. However, this strategy may also reduce specificity and increase false-positive results, particularly in BCG-vaccinated individuals, potentially leading to unnecessary preventive treatment. Current TBNET recommendations favours IGRA-based screening in BCG-vaccinated patients because prior BCG vaccination may affect TST specificity. Furthermore, serial IGRA testing during TNF-α inhibitor therapy has been suggested for selected high-risk individuals, although evidence supporting its clinical benefit remains limited. [4]

The optimal screening strategy in patients scheduled to receive immunosuppressive therapy remains a matter of ongoing debate. The current Dutch national guideline [15] reflects these insights and recommends a structured, risk-based approach, although this recommendation is primarily based on expert opinion rather than robust comparative outcome data. All candidates for immunosuppressive therapy first undergo a chest X-ray to evaluate for signs of active or previous TB disease. This is followed by a standardized risk assessment using a questionnaire covering medical history, symptoms, country of origin, living conditions, travel history, prior TB exposure, and previous screening or treatment for TB. Based on this evaluation, three pathways are outlined:

• *Very high risk***:** applies to individuals with a history of untreated or incompletely treated active TB or TBI. In these cases, preventive treatment is recommended without further testing, as the likelihood of false-negative results is high and the potential consequences of untreated or inadequately treated infection outweigh the risks of empirical therapy.
• *Low risk:* no screening with IGRA or TST is required, and immunosuppressive therapy can be initiated immediately in individuals born in the Netherlands after 1960 who have never had active TB or TBI, do not work in healthcare, have no contact with high-risk groups (such as people with alcohol or drug dependence, refugees, or homeless individuals), and have never traveled for more than three months to countries with a TB incidence exceeding 50 per 100,000 population.
• *Elevated risk*: in all other cases, dual testing with both TST and IGRA is recommended.

### 3.5 Treatment of paradoxical reactions during tuberculosis therapy

Several case reports have reported favorable outcomes with infliximab in patients with severe paradoxical reactions to anti-tuberculous therapy that were refractory to corticosteroids [16], particularly in cases of tuberculous meningitis with severe paradoxical neurological deterioration [17]. While data on the risk of tuberculosis recurrence during re-exposure to infliximab remain limited, no relapses have been reported in comparable published cases [18]. Clinicians should nevertheless remain vigilant for signs suggestive of tuberculosis relapse during follow-up, as distinguishing recurrent infection from a paradoxical inflammatory response may be challenging and has important therapeutic implications.

## 4 Learning points

This case illustrates the considerable risk of tuberculosis in patients receiving TNF-α inhibitor therapy and highlights the limitations of relying on a single negative screening test. Although current international recommendations vary, combined IGRA and TST testing may improve the detection of latent tuberculosis infection in selected high-risk populations, although evidence demonstrating improved long-term clinical outcomes remains limited. This dual testing strategy is currently recommended in Dutch national guidelines for patients at risk of TBI [15], though implementation is not yet consistent.

In immunocompromised populations, both IGRA and TST have reduced sensitivity, and indeterminate IGRA results occur more frequently due to impaired immune responses. These results should not be dismissed, as they are associated with a substantially higher risk of progression to active TB compared to true negative results. Careful interpretation and repeated, risk-based screening are therefore essential during long-term immunosuppression.

Importantly, TB risk is not static. Patients from or traveling to high-incidence countries require repeated assessment during long-term immunosuppressive therapy, taking into account travel history, exposure, and clinical context. Such a dynamic, risk-based approach is essential to avoid missed diagnoses and to improve patient outcomes.

## CRediT authorship contribution statement

**Aurelie J.F.Schroë:** Writing – original draft. **Marijke Rutten:** Writing – review & editing. **Sami O.Simons:** Writing – review & editing.

## Informed consent

Both written and verbal informed consents were obtained for the patient for publication of this case report.

## Ethical Statement

This case report was conducted in accordance with the ethical standards of Maastricht University Medical Center+. Both written and verbal informed consents were obtained for the patient for publication of this case report and accompanying clinical details. All procedures performed were in accordance with the Declaration of Helsinki and local ethical guidelines.

## Declaration of competing interest

The authors declare that they have no known competing financial interests or personal relationships that could have appeared to influence the work reported in this paper.

## Data Availability

The authors declare that data supporting the findings of this study are available within the article.
